# A Chapter on Medical Ethics

**Published:** 1882-04

**Authors:** 


### EDITORIAL DEPARTMENT.

A Chapter on Medical Ethics.—At the last meeting of the American Medical Association at Richmond, Dr. E. S. Dunster, of Michigan, offered the following resolution in the section on Practice of Medicine, Materia Medica, and Physiology :

*Resolved*, That the spirit of the code of ethics forbids a physician from prescribing a remedy controlled by a patent, copyright, or trade-mark. This, however, shall except a patent upon a process of manufacture or machinery, provided such patent be not used to prevent legitimate competition ; and shall also except the use of a trade-mark used to designate a brand of manufacture, provided that the article so marked be accompanied by working formulæ duly sworn to, and also by a technical scientific name, under which any one can compete in the manufacture of the same.

This resolution was adopted by the section and referred to the general session of the association, where it was, on motion of Dr. Toner, of the District of Columbia, referred to the Judicial Council, which body is expected to report upon it at the meeting of the association at St. Paul, in June next.

Any one familiar with the code of ethics of the association, which, until recently, was supposed to control the action of members of the profession toward each other, and to guide them in all their duties to the profession at large, knew that the code expressly condemned "the giving of certificates attesting the efficiency of patent or secret medicines," as "*reprehensible*," and by implication "derogatory to professional character." In the new code recently adopted by the Medical Society of the State of New York, which has been looked upon in many places as giving physicians too much latitude in their professional relations to each other and to the public, this condemnation is equally emphatic and much more comprehensive in its scope. It reads thus :

"It is equally derogatory to professional character, and opposed to the interests of the profession, for a physician to hold a patent for any surgical instruments or medicine, or to prescribe a secret nostrum, whether the invention or discovery be the exclusive property of himself or others.

"It is also reprehensible for physicians to give certificates attesting the efficacy of patented medical or surgical appliances, or of patented, copyrighted, or secret medicines, or of proprietary drugs, medicines, wines, mineral waters, health resorts, etc."

It was generally known at the Richmond meeting of the association, that Messrs. Parke, Davis & Co., the great manufacturing pharmacists of Detroit, were active in endeavoring to obtain the passage of the resolution containing this expression of condemnation of a practice generally regarded as vicious in its tendencies, if not in its immediate results. Since that time this firm and its methods have been at various times assailed in a more or less open manner in certain medical journals. It has been generally believed among medical journalists that these attacks were made at the instigation of certain manufacturers of patented or trade mark preparations, which are more or less used by physicians in their prescriptions. For ourselves, we believe it is at least unscientific, not to use the harsher term used in the code, for a physician to prescribe a remedy of whose composition he knows little, of whose preparation he knows less, and whose action he can only measure by the clinical results of its application, a confessedly very insecure ground to stand on. With such a medicine there can be no question of determining its physiological action. The manufacturer prepares the mixture, adds to the supposed effective constituent certain other things to render it more palatable, sticks a red-line label on the bottle, marked "For Consumption—one teaspoonful three times a day," wraps up the bottle in a circular containing certificates of the efficacy of the remedy in various diseases, from "the eminent Dr. John Johnston, the renowned Professor Wyllys Wyllyams, and a score of others." He then sends bottles of various sizes to the physicians, who are expected to yield their own judgment to the authority of the signers of the certificate, who may, or may not be "eminent" or "renowned," as the circular and the oily tongue of the colporter assert. This evil has within recent years grown to such an enormous degree that, in our judgment, the resolution of Dr. Dunster was not presented at all too soon, and the fact that it was so promptly adopted by the Section on Practice of Medicine and referred to the general session for adoption, showed that the members of the American Medical Association thought so likewise. From the constitution of the Judicial Council, we are led to expect a report at the St. Paul meeting which will definitely settle the meaning of the section of the code which we have above quoted.

We referred above to the attacks that have been made upon the house of Parke, Davis & Co. by interested parties. Until very recently these animosities, when they have cropped out, have been limited, on the one or other side, to the parties supposed to be pecuniarily interested. In the February number of the *New England Medical Monthly*, however, an article appears, the responsibility of which is assumed by Dr. Horatio R. Bigelow, of Washington, which, while making a violent attack upon Messrs. Parke, Davis & Co., at the same time makes an unwarranted and indecent charge against the American Medical Association. So long as the quarrel was between Parke, Davis & Co., on one side, and the proprietors of patent, secret and trade-mark preparations on the other, we were not concerned, although our sympathies were with the former. When, however, the dignity of medical journalism is lowered by the admission of such an abusive article as Dr. Bigelow's, and when, in addition, the attempt is made to besmirch the honor and integrity of the members of the American Medical Association, and through them, of the whole medical profession—or that part, at least, which looks with disfavor upon the prescribing of quack medicines, we are obliged to protest. The insinuation is made that Parke, Davis & Co., "dispensed free cigars, free rum, and free everything" at Richmond, as a bribe to the recipients of these attentions to vote for the adoption of the resolution at the head of this article. As the resolution was adopted in the section where it was presented, the inference is that those who voted for the resolution were bribed or "influenced" by the courtesies received from their entertainers. Such a gratuitous insult to the profession comes from one who himself claims to be a physician in good standing, and is printed in a presumably reputable medical journal without a word of protest from the editor. It is said, and affidavits are published to prove the statement, that the number of the journal containing Dr. Bigelow's article was circulated to the extent of 60,000 copies. We should like to know how those gentlemen who voted for the resolution at Richmond felt, when they read the article in which they are so incontinently slapped in the face.

We have no doubt that the honest sense of the medical profession of this country will appreciate at its proper importance this attempt to besmirch the character of their representatives in the National Association, and hope that the local societies in every State will instruct their delegates to the St. Paul meeting to use every endeavor to secure the adoption of such measures as will tend to put an end to the patent medicine humbug, with its undignified certificate system and other similar accessories. The scientifically educated physician needs no such meretricious aids in the treatment of disease. He should be, and is, more competent to select and combine the remedies suitable in a given case, than any trade druggist or nostrum compounder in the country, even though the latter be endorsed by all the " exalted " ones, who, we are told by Dr. Bigelow, " have left their impress upon medical practice," and whose " books are the student's treasures." While acknowledging their eminence, and honoring them for the good they have done, we would like to suggest that even the young practitioner does his own thinking largely nowadays, and his reverence for his teachers is subordinated to the demands of reason, experience, and common sense.

